# Care Trajectories of People With Mood Disorders in Quebec Using a Sequence Analysis Method

**DOI:** 10.1002/brb3.71260

**Published:** 2026-02-16

**Authors:** Marc Henri N'Guessan, Matea Belan, Christian Roger Clav Kouakou, Maude Laberge, Thomas Gilbert Poder

**Affiliations:** ^1^ Centre d'études et de recherches sur le développement international Université Clermont Auvergne Clermont‐Ferrand France; ^2^ Département d'économique, École de gestion Université de Sherbrooke Sherbrooke QC Canada; ^3^ Vice‐décanat à la recherche et aux études supérieures, Faculté de Médecine Université de Sherbrooke Sherbrooke QC Canada; ^4^ Centre de recherche de l'IUSMM CIUSSS de l'Est de l'Île de Montréal Montréal QC Canada; ^5^ Département de médecine sociale et préventive, Faculté de médecine Université Laval Québec QC Canada; ^6^ Département de gestion, d'évaluation et de politique de santé École de santé publique de l'Université de Montréal Montréal QC Canada

**Keywords:** care trajectories, sequence analysis, health services utilization, healthcare planning, mood disorders

## Abstract

**Purpose:**

The analysis of care trajectories for chronic diseases has gained increasing importance, particularly for mental health conditions that are often neglected despite their alarming prevalence. This study aimed to identify and describe care trajectories of Quebec adults with self‐reported mood disorders.

**Method:**

We used data from the TorSaDE cohort, which links data from the Canadian Community Health Surveys (CCHS) with administrative data from Quebec's health insurance board Régie de l'assurance maladie du Québec (RAMQ) over a 21‐year period (1996–2016). Sequence analysis (SA) was used for 4421 Quebec adults who self‐reported mood disorders within a 7‐year follow‐up period.

**Finding:**

SA revealed three distinct user groups: Low healthcare users (Type 1, *n* = 2714), moderate users with diverse services (Type 2, *n* = 1120), and moderate users with high psychiatric service use (Type 3, *n* = 587). Average healthcare costs over 7 years varied significantly between trajectory types, ranging from $94,434 for low users to $230,899 for moderate users.

**Conclusion:**

Results show that the burden of mood disorders is unevenly distributed across care trajectories: a small vulnerable group accounts for a disproportionate share of specialized healthcare use, while the majority relies only marginally on the public system. These findings highlight the heterogeneity of patients with mood disorders and underscore the need for differentiated, profile‐specific approaches to healthcare planning rather than uniform treatment strategies.

## Graphical abstract

Sequence analysis (SA) revealed three distinct user groups: low healthcare users (Type 1, *n* = 2714), moderate users with diverse services (Type 2, *n* = 1120), and moderate users with high psychiatric service use (Type 3, *n* = 587).

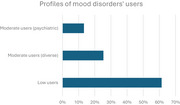

## 1 Introduction

Mood disorders (MDs) are a category of mental illnesses primarily characterized by emotional disturbances that manifest as persistent and pervasive feelings of depression, mania, or both. Major depressive disorder, affecting approximately 5.4% of the Canadian population, and bipolar disorder, which affects nearly 1%, are the two main conditions classified under MDs (Association canadienne pour la santé mentale 2021). These conditions represent a major public health challenge because of their chronicity, disabling consequences, and the significant burden they impose on health care systems.

The global prevalence of mental health problems is alarming, and mental illness has been increasingly recognized as an urgent but often neglected societal issue (Datta and Petticrew, 2013; Nations Unies 2017). In Canada, the Canadian Mental Health Association reports that one in five individuals experiences a mental health problem in any given year, and by the age of 40, nearly half of Canadians will have experienced a mental illness (Association canadienne pour la santé mentale 2021). The economic burden is also substantial: in 2011, the direct costs of mental illness—including medical care, hospitalization, pharmaceuticals, and community and social services—were estimated at CAD 42.3 billion (Commission de la santé mentale du Canada 2017).

Individuals living with MDs consistently demonstrate higher levels of health service use than people without MDs. National evidence shows, for example, that individuals with a mood disorder have a threefold higher risk of frequent emergency department use compared with those without such disorders (Fehlmann et al., 2022). Complementary findings indicate that young people with mental disorders—including MDs—also exhibit significantly higher rates of hospitalizations, specialist consultations, and ambulatory care visits relative to their peers without mental disorders (Mitchell et al., 2022). Despite this overall elevated utilization, patterns remain highly heterogeneous: some individuals have minimal contact with the healthcare system, whereas others rely heavily on specialized psychiatric care (Kouakou et al., 2025). This variability underscores the importance of improving our understanding of the diversity of care trajectories among people living with MDs.

The concept of a care trajectory refers to the individual sequence of health care utilization over a defined period (Vanasse et al., 2018). Analyzing these trajectories enables identifying the most efficient ones, that is, those that minimize potentially inappropriate service use while maximizing health outcomes for patients. Such analysis is of critical importance, not only for policymakers, who can use the results to redesign and reorganize health services, but also for patients, by improving their overall experience within the healthcare system. Despite its importance, research on care trajectories in mental health remains relatively recent and methodologically underdeveloped. While some analytical methods have been proposed, there is still limited consensus on the optimal approaches to identify typical trajectories (Organisation mondiale de la Santé 2022).

This study contributes to this literature by applying sequence analysis (SA) to identify typical care trajectories among individuals living with mood disorders. Using data from the Care Trajectories–Enriched Data (TorSaDE) cohort, which links Canadian Community Health Survey (CCHS) data with Quebec administrative health data from 1996 to 2016, we characterized these trajectories and assessed their costs.

## 2 Methods

### 2.1 Data Sources and Cohort

The study population was drawn from the TorSaDE cohort, which links respondents from the CCHS residing in Quebec with administrative data from the Régie de l'assurance maladie du Québec (RAMQ). The present analysis included individuals who completed at least one cycle of the CCHS between 2007 and 2016 and provided consent for their survey data to be linked with RAMQ records.

The identification of participants with MDs relied on two potential approaches. The first approach was self‐reporting: respondents were asked in the CCHS whether they had been diagnosed by a health professional with a mood disorder such as depression, bipolar disorder, mania, or dysthymia. The second approach was diagnostic information from physicians, available through RAMQ medical billing data. For the purposes of this study, we adopted the self‐reporting approach. This decision was guided by several considerations.

First, self‐reported diagnosis captures the respondent's lived experience at the time of the CCHS interview and enables the use of other survey data provided by participants. Indeed, the study aims to analyze care trajectories from the perspective of the patient's lived experience. Self‐reporting was deemed more robust because it captures individuals who are conscious of their condition and live with its consequences daily. While the validity of the sample is not compromised, its definition is specific; the sample does not represent all individuals who have ever received a psychiatric billing code, but rather Quebec adults who recognize themselves as living with a MD.

Second, experts consulted for this project highlighted concerns regarding the validity of RAMQ diagnoses for identifying MD cases, as highlighted by other studies in the Quebec context (Kouakou et al., 2025; Dufour et al., 2022). Indeed, each medical visit in the RAMQ system is associated with a single diagnostic code; consequently, patients with multiple conditions may not have the MD diagnosis in the system if the health provider entered another condition for the visit. For instance, a patient with both MD and diabetes might have the visit recorded under diabetes. Hence, relying on RAMQ data could lead to underestimating MD prevalence. Conversely, short‐term or mild depressive episodes might inflate MD's estimates if used as the sole diagnostic criterion. Data‐entry errors are also possible.

Given these methodological limitations, the self‐reported measure was deemed more robust and more consistent with the objectives of analyzing care trajectories from the patient's perspective. As a result, all individuals who self‐identified as having been diagnosed with a mood disorder in the CCHS were included in the sample analyzed.

### 2.2 Study Population and Index Date

The population under study consisted of adults residing in Quebec who self‐reported a diagnosis of MD in the CCHS and consented to the linkage of their survey data with the RAMQ database. Inclusion criteria were (i) age 18 years or older at the time of the CCHS interview; (ii) completion of at least one CCHS cycle between 2007 and 2016; (iii) self‐reported diagnosis of MD by a health professional, including depression, bipolar disorder, mania, or dysthymia; and (iv) availability of linked administrative follow‐up data. Individuals who did not consent to data linkage or who lacked valid identifiers for RAMQ linkage were excluded from the cohort.

**FIGURE 1:**
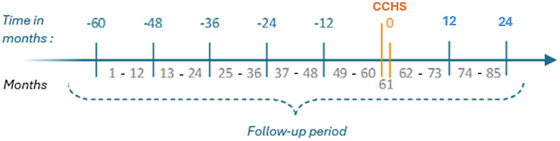
Follow‐up period for defining care trajectories for sequence analysis.

The index date was defined as the date of the CCHS interview at which the respondent reported a MD diagnosis. This index serves as the temporal anchor for constructing trajectories of service utilization. The follow‐up covered a period of seven years, namely the five years preceding the index date and the two years following it. For example, if an individual reported a MD on October 21, 2009, the observation extended from October 21, 2004, to October 21, 2011. To preserve monthly granularity, the total observation period consisted of 85 months: 84 consecutive months plus the additional month corresponding to the index date itself (Figure 1).

The rationale for adopting this timeframe was threefold: (i) to ensure a sufficiently long retrospective horizon to capture diverse trajectories of service use prior to the reported diagnosis; (ii) to allow for prospective follow‐up to observe post‐index patterns; and (iii) to maximize the use of available administrative data while balancing feasibility and interpretability.

### 2.3 Outcomes and Covariates

The construction of the database for SA required the identification of a common set of health services utilization variables derived from RAMQ administrative data. These variables were harmonized into categories suitable for trajectory modeling. Variables of interest included (i) hospitalizations, (ii) emergency department (ED) visits, (iii) outpatient consultations with a psychiatrist, (iv) outpatient consultations with a medical specialist other than psychiatry, and (v) outpatient consultations with a general practitioner (GP). To capture trajectories in a discrete‐state framework, we defined five mutually exclusive states following a hierarchical priority order:

1. Hospitalization or emergency department (ED) visit;
2. Consultation with a psychiatrist;
3. Consultation with another medical specialist (non‐psychiatrist);
4. Consultation with a general practitioner (GP);
5. No service (null utilization).

Given the relatively low frequency of hospitalizations, an average of 5.38 ± 7.60 per individual over the seven‐year follow‐up, we combined hospitalizations and ED visits into a single category. This decision was justified by the sparse distribution of hospitalization events (approximately one hospitalization every 18 months on average), which would otherwise result in excessive null states and reduce the efficiency of the SA. Despite this aggregation, hospitalizations and ED visits were common events at the cohort level: 81.2% of individuals had at least one hospitalization, and 95.4% had at least one ED consultation during the observation window.

In addition to health services utilization outcomes, a set of socio‐demographic and health‐related variables from the CCHS were used as covariates in regression models to explore determinants of trajectory membership. These included age, sex, household income, marital status, household size, educational attainment, and self‐rated health and stress. These covariates were selected for their theoretical and empirical relevance to patterns of health‐services utilization among individuals with MDs (Kouakou et al., 2025; Dufour et al., 2022).

### 2.4 Statistical Analyses

Sequence analysis is a statistical technique used to study ordered successions of states or events over time. Rather than treating observations as independent, SA considers the temporal order and interdependence of events within individual trajectories. Each individual trajectory—also called a sequence—represents the chronological succession of distinct states, allowing for the identification of similarities and differences across individuals through common subsequences.

In this study, we defined trajectories as monthly sequences of health service utilization states observed over an 85‐month follow‐up period, corresponding to 5 years before and 2 years after the index date. Each sequence was thus composed of 85 elements, each representing 1 month and the most prioritized state experienced during that period. When multiple types of contacts occurred within the same month, the highest‐priority state was retained (i.e., from hospitalization or ED visit to no service use). For instance, if a person was hospitalized and also consulted a psychiatrist in the same month, the recorded state for that month was “hospitalization.”

Once the sequences were constructed, pairwise comparisons were performed to assess their similarity. We applied the Optimal Matching Algorithm (OMA), a dynamic programming method that quantifies the distance between two sequences by determining the minimum number of elementary operations—insertions, deletions, or substitutions—required to transform one sequence into another. Insertions and deletions (indels) were each assigned a unit cost of one, while substitutions were assigned a cost of two, reflecting their equivalence to one deletion plus one insertion. The greater the cumulative transformation cost, the more dissimilar the two sequences. Table 1 illustrates this calculation for two theoretical sequences composed of 6 measurement times. The algorithm seeks to minimize the total cost to transform Sequence B into Sequence A. In this example, the total calculated cost is 6, derived from the sum of necessary operations: one suppression (at time 3), one insertion (at time 4), and two modifications (at times 5 and 6).

**TABLE 1:** Transformations’ operations and costs for sequences comparison.

Sequence/State	1	2	3	4	5	6
A	H	H	—	A	A	U
B	H	H	U	—	U	H
Transformation	E	E	S	I	M	M
Cost	0	0	1	1	2	2

**Abbreviations for columns 1 to 6**: H, hospitalization; A, consultation with another medical specialist; U, Emergency department visit; E, Equivalent, no cost assigned; I, insertion (cost = 1); M, modification (cost = 2); S, suppression (cost = 1).

After computing the distance matrix for all pairs of sequences, we conducted cluster analysis to identify distinct patterns of healthcare utilization trajectories. We employed Ward's hierarchical agglomerative algorithm, which iteratively merges sequences into clusters in a way that minimizes within‐cluster variance and maximizes between‐cluster heterogeneity. The optimal number of clusters was determined based on internal quality criteria (e.g., average silhouette width, pseudo‐*F* statistic) and the interpretability of resulting groups. The hierarchical structure of clustering results was visualized using dendrograms, facilitating the identification of meaningful trajectory types.

All sequence analyses were conducted in R using the TraMineR package, which offers advanced tools for constructing, visualizing, and comparing sequences. Stata was used for data management and descriptive analyses preceding sequence construction.

### 2.5 Cost Analysis

Following the identification of care trajectories through SA, we conducted a cost analysis from the perspective of the Quebec public healthcare system. We focused on direct medical costs available through RAMQ and related provincial databases. The cost categories included (i) hospitalizations, (ii) same‐day surgeries (available from 2010 onward), (iii) ED visits, (iv) physician visits (GP and specialists), and (v) pharmaceutical expenditures for individuals covered under the public drug insurance plan (i.e., those aged ≥ 65 years or with low income).

Hospitalization and same‐day surgery costs were calculated using the relative intensity level of resource use (NIRRU) provided by the Ministry of Health and Social Services. The NIRRU reflects the relative resource consumption for each episode. We multiplied the NIRRU assigned to each hospitalization or surgical procedure by the standardized monetary value of one NIRRU in the reference year (2019). This corresponded to CAD 6,026.06 for a hospitalization, CAD 1,368.30 for a same‐day surgery, and CAD 355.60 for an ED visit. For ED visits, where individual‐level NIRRUs were not available, each visit was assigned the standardized ED cost.

Physician visits were valued using the amount billed to RAMQ for each act performed, expressed in current dollars of the billing year and then converted into 2019 constant dollars using the Consumer Price Index (CPI). Pharmaceutical expenditures included dispensing fees and the cost of drugs reimbursed by the public plan; these were also converted into 2019 constant dollars using the CPI. This costing framework allowed us to assign standardized 2019 values across all service categories and to compute total and category‐specific costs per patient trajectory.

## 3 Results

### 3.1 Participants’ Characteristics

**FIGURE 2:**
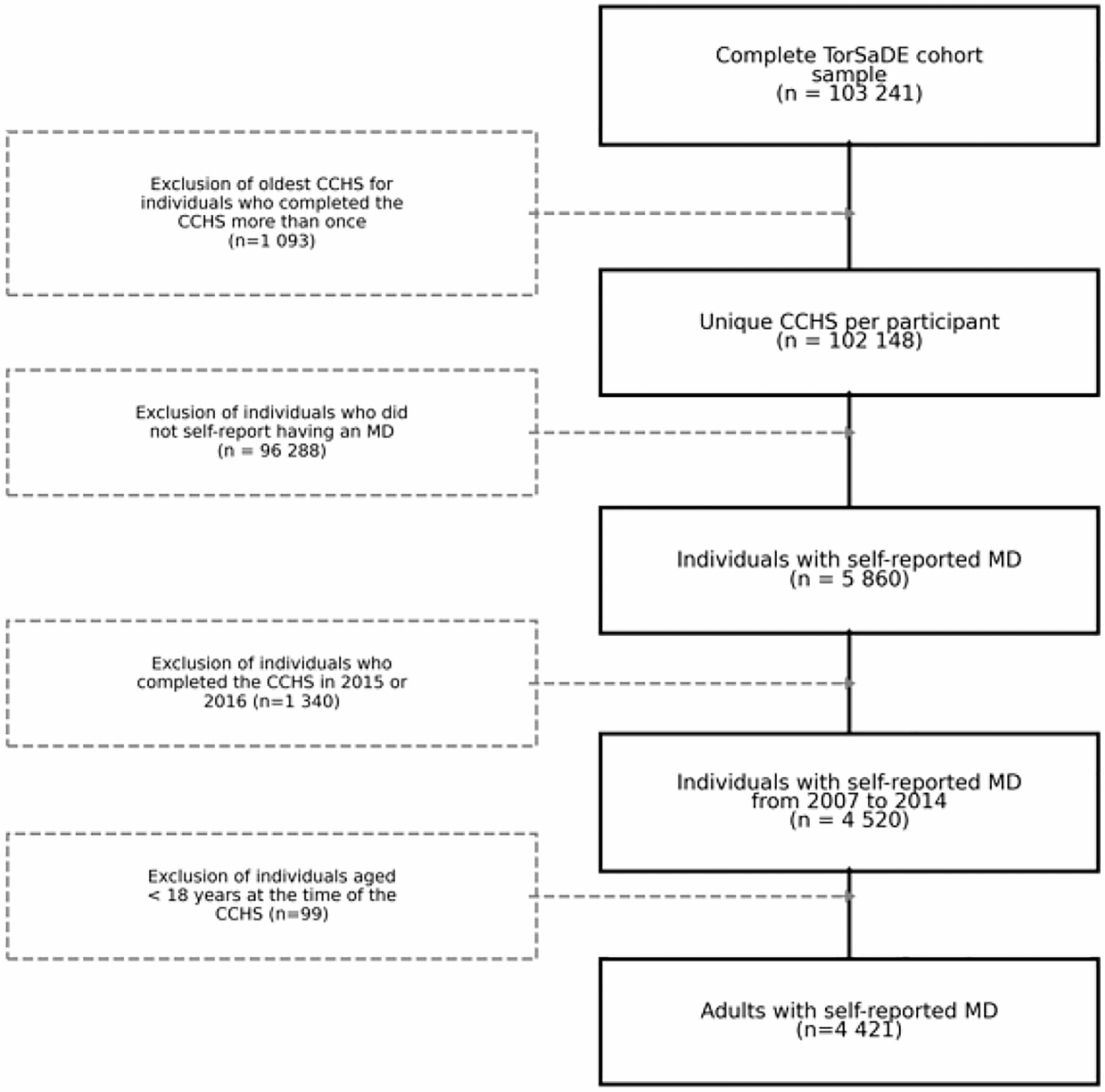
Participant flow diagram.

*Sample*. The participant flow diagram is shown in Figure 2. The TorSaDE cohort contains 103,241 CCHS entries. After retaining the most recent CCHS entry for individuals with multiple responses (*n* = 1093), there were 102,148 unique CCHS entries. Of these, 5860 respondents self‐reported a mood disorder. Excluding respondents interviewed in 2015–2016 (insufficient post‐index follow‐up) and those <18 years at interview yielded a final sample of 4421 adults with self‐reported MD (CCHS 2007–2014).

*Agreement between self‐reported and administrative diagnoses*. Within the 2007–2014 cohort period, 9,765 individuals had at least one RAMQ medical encounter carrying an MD diagnosis code. Among these, 71.4% (6,969/9,765) did not self‐report MD on the CCHS. Conversely, among adults who did self‐report MD, 36.8% (1625/4,421) had no MD diagnosis code in RAMQ during the seven‐year observation period (Table 2).

**TABLE 2:** Cross‐tabulation of self‐reported vs. RAMQ diagnosis.

RAMQ diagnosis data			
CCHS self‐reported	No MD diagnosis code (0)	Presence of MD diagnosis code (1)	Total
No self‐reported MD (0)	62,748	6969	69,717
Self‐reported MD (1)	1625	2796	4421
Total	64,373	9765	74,138

*Sociodemographic profile*. In the self‐reported MD sample (*n* = 4421), the mean age was 50.7 ± 15.7 years, and 60.8% were women. Body mass index averaged 27.2 kg/m^2^, with higher proportions in obesity classes I–III than in the general TorSaDE population. Marital status skewed away from marriage (28.1% married; 31.6% single/never married). Household and respondent incomes were lower than in the general cohort, with smaller shares in the upper income brackets.

*Perceived stress and health*. Most participants reported stress in daily life (80.9%) and at work (83.2%). Despite this, self‐rated mental health and general health were often positive: 67.8% and 65.0%, respectively, describing their status as good to excellent.

*Health‐services use in the 7‐year period*. Utilization was higher in the MDs sample than in the overall cohort, especially for psychiatry and general practice. Median (IQR) contacts were: hospitalizations 3 (1–7); ED visits 18 (8–36); GP visits 104 (62–166); other specialists 63 (31–117); and psychiatrist 3 (0–32). Summary descriptives for the MDs sample versus the full TorSaDE cohort are presented in Table 3.

**TABLE 3:** Baseline characteristics.

Characteristics	MD Sample (*n* = 4421)	TorSaDE Sample (*n* = 80,651)	*p*‐value
**Age, years**	50.7 ± 15.7	48.2 ± 20.2	< 0.001
**Female, %**	60.8%	50.6%	< 0.001
**Body mass index, kg/m^2^**	27.2 ± 6.04	25.6 ± 5.18	< 0.001
Underweight (BMI < 18)	1.76%	2.88%	< 0.001
Normal weight (18 ≥ BMI < 25)	37.6%	46.8%	< 0.001
Overweight (25 ≥ BMI < 30)	30.1%	30.3%	0.7574
Obese class I (30 ≥ BMI < 35)	16.1%	11.2%	< 0.001
Obese class II (35 ≥ BMI< 40)	5.52%	2.80%	< 0.001
Obese class III (BMI ≥ 40)	8.99%	6.00%	< 0.001
**Education level, %**			
Unknown	5.86%	7.35%	0.0148
Secondary not completed	21.5%	22.7%	0.0007
Secondary school diploma	14.0%	12.7%	0.0742
Professional diploma or CEGEP	25.6%	22.3%	< 0.001
Bachelor's degree	21.0%	21.5%	0.0109
Graduate university studies	12.1%	13.5%	< 0.001
**Marital status, %**			
Married	28.1%	35.8%	< 0.001
Common‐law	18.6%	20.8%	< 0.001
Widowed	4.98%	4.86%	0.0522
Separated	4.15%	2.54%	< 0.001
Divorced	12.6%	6.02%	< 0.001
Single/Never married	31.6%	30.0%	0.0098
**Annual household income, %**			
Less than $20,000	21.9%	10.2%	< 0.001
$20,000 to less than $40,000	25.1%	21.1%	< 0.001
$40,000 to less than $60,000	19.2%	19.6%	0.3936
$60,000 to less than $80,000	12.8%	16.2%	< 0.001
$80,000 and more	21.1%	32.9%	< 0.001
**Annual respondent income, %**			
Less than $20,000	44.6%	32.2%	< 0.001
$20,000 to less than $40,000	28.7%	30.1%	0.5720
$40,000 to less than $60,000	14.7%	19.6%	< 0.001
$60,000 to less than $80,000	7.63%	10.1%	< 0.001
$80,000 and more	4.39%	8.02%	< 0.001
**Country of birth, %**			
Canada and North America	91.4%	86.1%	< 0.001
Central and South America	1.87%	3.20%	< 0.001
Europe	3.44%	4.62%	< 0.001
Asia and Oceania	1.71%	3.25%	< 0.001
Africa	1.54%	2.82%	< 0.001
**Perceived stress – Daily life, %**			
Not at all stressed	6.53%	14.0%	< 0.001
Not very stressed	12.6%	21.3%	< 0.001
A bit stressed	34.2%	38.8%	< 0.001
Quite a bit stressed	34.5%	22.4%	< 0.001
Extremely stressed	12.2%	3.57%	< 0.001
**Perceived stress – Work, %**			
Not at all stressed	5.92%	8.89%	< 0.001
Not very stressed	11.0%	15.6%	< 0.001
A bit stressed	31.8%	39.4%	< 0.001
Quite a bit stressed	38.0%	30.7%	< 0.001
Extremely stressed	13.4%	5.50%	< 0.001
**Perceived general health, %**			
Poor	9.59%	2.01%	< 0.001
Fair	25.5%	8.23%	< 0.001
Good	36.5%	30.4%	< 0.001
Very good	22.6%	36.3%	< 0.001
Excellent	5.87%	23.1%	< 0.001
**Perceived mental health, %**			
Poor	7.92%	0.56%	< 0.001
Fair	24.9%	3.39%	0.0462
Good	41.6%	20.5%	< 0.001
Very good	19.1%	35.8%	< 0.001
Excellent	6.56%	39.8%	< 0.001
**Health‐related quality of life index (HUI‐3)** ^β^ **, score**	0.68 ± 0.30	0.88 ± 0.18	< 0.001
**Health service use (mean ± SD; median [IQR])**			
Hospitalizations	5.38 ± 7.61 3 [1 – 7]	3.16 ± 4.81 2 [0 – 4]	< 0.001 <0.001^α^
Emergency department visit	28.5 ± 35.4 18 [8 – 36]	17.2 ± 23.7 10 [4 – 22]	< 0.001 < 0.001^α^
Consultation with a psychiatrist	40.4 ± 101.8 3 [0 – 32]	5.01 ± 33.5 0 [0 – 0]	< 0.001 < 0.001^α^
Consultation with another medical specialist	92.9 ± 112.5 63 [31 – 117]	71.1 ± 90.9 44 [18 – 92]	< 0.001 < 0.001^α^
Consultation with a GP	127.5 ± 103.2 104 [62 – 166]	75.9 ± 76.3 55 [26 – 101]	< 0.001 < 0.001^α^
**Proportion with at least one consultation, %**			
Hospitalizations	81.2%	68.2%	<0.001
Emergency department visit	95.4%	88.5%	<0.001
Consultation with a psychiatrist	59.8%	14.8%	<0.001
Consultation with another medical specialist	99.6%	98.1%	<0.001
Consultation with a GP	99.7%	99.3%	0.1517

^α^ Comparison performed by Wilcoxon *t*‐test, for single sample.

^β^ Score not available for individuals who completed the 2011–2012 cycle of the CCHS (*n* = 19,966).

### 3.2 Sequence Analysis

The sequence analysis (SA) revealed substantial heterogeneity across care trajectories. Among the 4421 respondents, 4407 unique sequences were identified, corresponding to a concentration rate of 99.6%. Only 16 individuals shared an identical trajectory, either marked by the absence of service use throughout the follow‐up (*n* = 14) or by a single consultation with a GP (*n* = 2). On average, individual trajectories were composed of 41.2 episodes (SD = 14.0), where an episode corresponds to a consecutive subsequence of identical states within a respondent's trajectory. In other words, this measure reflects the number of distinct consecutive segments (e.g., successive months spent in the same type of care state), rather than the total number of months in each state. The most frequent type of consecutive subsequence was no service use, with an average duration of 15.7 consecutive months (SD = 5.96), followed by consultations with a general practitioner (10.4 ± 5.74 months) and consultations with other medical specialists (8.2 ± 5.84 months). Psychiatric consultations were the least frequent, lasting on average 2.7 ± 5.65 consecutive months (0 [0–2]).

**FIGURE 3:**
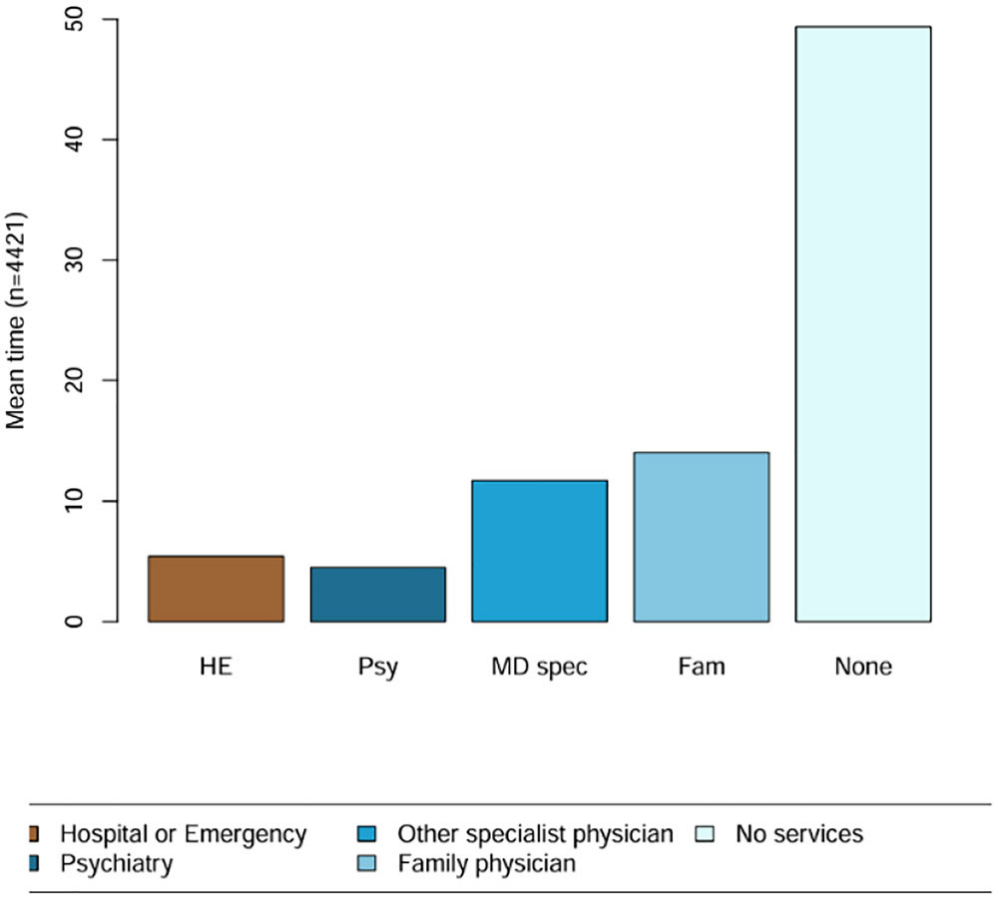
Mean time spent (in months) per state.

As illustrated in Figure 3, respondents spent on average 50 months—nearly 59% of the 85‐month sequence—without any contact with the healthcare system. Psychiatric consultations remained the least frequent type of service use (mean use of 4.5 months; 5.3%), followed by hospitalizations and emergency visits (5.4 months; 6.4%).

**FIGURE 4:**
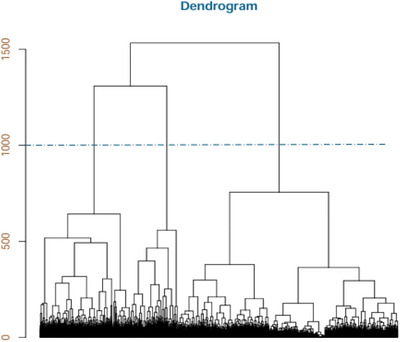
Dendrogram of the sequences clustering.

Once constructed, sequences were compared using OMA, from which distance calculations were used to perform the cluster analysis. To determine the optimal number of clusters to retain, two visual aids were produced. The first, a dendrogram (Figure 4), illustrates the distances between individual sequences (at the bottom of the figure) and the distances between progressively merged clusters of similar sequences. Each plateau represents a grouping of trajectories. To identify the optimal number of clusters, a horizontal line is drawn across the diagram at the point where the greatest vertical distance between plateaus is observed (as indicated by the dashed line in Figure 4).

**FIGURE 5:**
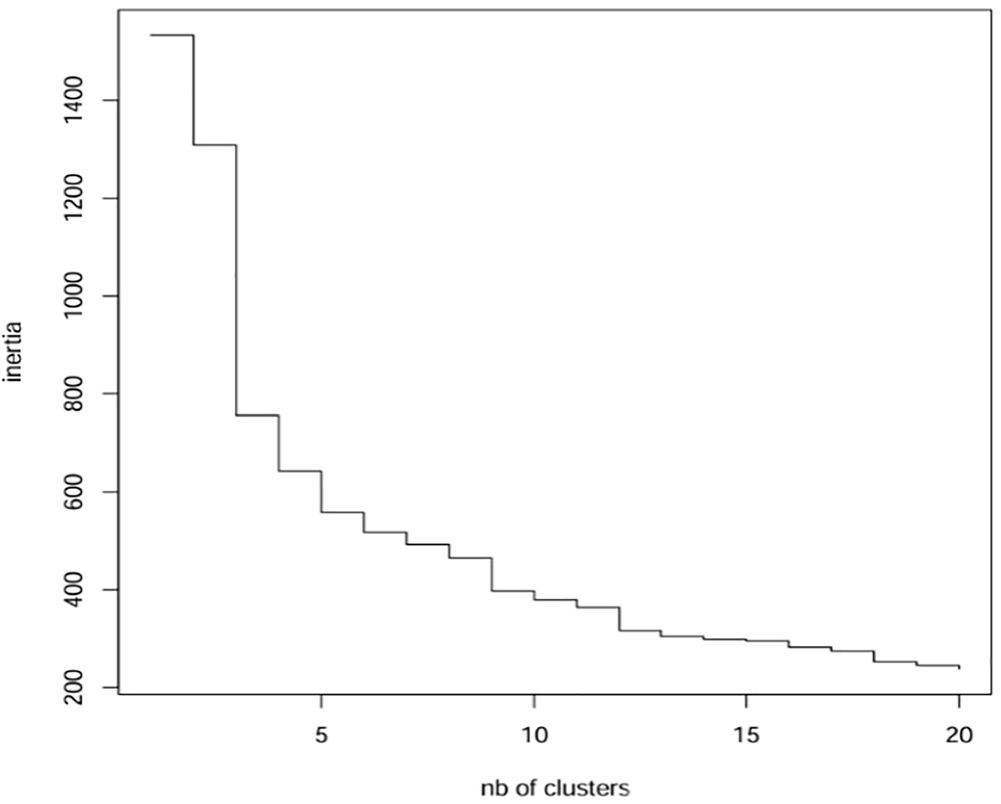
Inertia jump plot.

The second visual aid displays an inertia jump plot (Figure 5). This graph presents inertia on the y‐axis, which represents the differences between clusters in terms of variance and covariance. The objective was to select a number of clusters maximizing the differences between groups while minimizing the variability within each group. As shown in Figure 5, there is a sharp increase in inertia when moving from three to two clusters, whereas the increase is smaller when moving from four to three clusters. These observations, combined with the insights obtained from the dendrogram, suggest that the optimal number of clusters in our sample is three.

**FIGURE 6:**
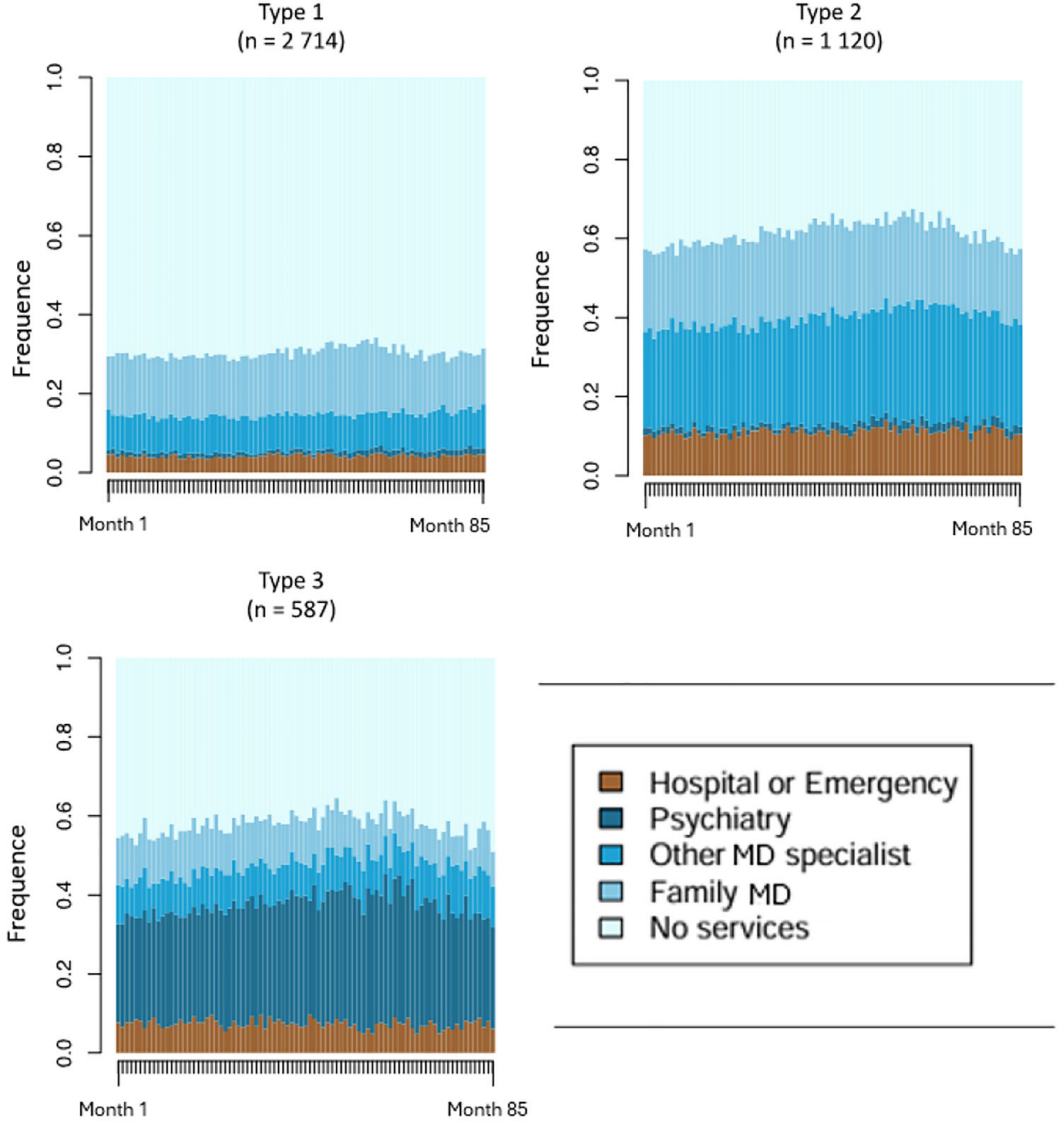
Monthly distribution of respondents by type of contact with the health care system (states).

Figure 6 presents the monthly frequency of health service contacts per respondent. A clear difference in health service utilization can be observed across the three groups identified through the cluster analysis. Type 1 (*n* = 2714) is the largest group. Individuals in this group had very limited contact with the health care system over the 85‐month follow‐up period, with only about 30% having any contact at all. Type 2 (*n* = 1120) appears to have a higher frequency of hospitalizations and consultations with general practitioners or non‐psychiatrist specialists. Finally, Type 3 (*n* = 587) consists mainly of high users of specialized psychiatric services.

A comparison of descriptive characteristics across the three user types (Table A1, Appendix) shows that a higher proportion of individuals in Type 3 had completed graduate‐level university education (16.3% vs. 10.9% and 11.7% for Types 2 and 1, respectively), were single or never married (42.2% vs. 19.9% and 33.6%), earned less than $20,000 per year (52.3% vs. 45.3% and 42.7%), and reported their mental health as poor (14.0% vs. 7.16% and 6.97%) or fair (36.0% vs. 23.5% and 23.1%).

### 3.3 Health Care Costs by Trajectory

Mean cumulative costs over 7 years differed markedly by trajectory (Table 4). Type 1 incurred the lowest costs (mean CAD 94,434 ± 309,190), reflecting minimal service use. Type 2 had the highest costs (CAD 230,899 ± 514,675), largely due to frequent hospital and specialist use. Type 3 costs averaged CAD 191,248 (±519,904). The cost differences between Type 2 versus Type 1 and Type 3 versus Type 1 were statistically significant (*p* < 0.001), whereas Type 2 versus Type 3 did not differ significantly. Thus, trajectories with more intensive care were associated with substantially higher expenditures.

**TABLE 4:** Mean total costs per individual (CAD) by type of user defined by sequence analysis.

Cost category (mean, standard deviation, median, interquartile)	Type 1 (*n* = 2714)	Type 2 (*n* = 1120)	Type 3 (*n* = 587)	*p*‐value
Hospitalizations	$74,347.05 ± 305 664.00 $0 [0–3522.83]	$185,279.20 ± 512,424.50 $6702.18 [0–120,199.40]	$153,057.00 ± 516 841.20 $0 [0–38 363.02]	2 vs. 1: < 0.001 3 vs. 1: < 0.001 3 vs. 2: 0.253
Outpatient surgery	$7725.47 ± 35,737.59 $0 [0–0]	$9818.80 ± 33,610.94 $0 [0–291.45]	$5564.68 ± 26,303.01 $0 [0–0]	2 vs. 1: 0.090 3 vs. 1: 0.513 3 vs. 2: 0.038
Emergency department visits	$2672.63 ± 3538.84 $1422.40 [355.60–3556.00]	$7583.81 ± 9495.63 $4978.40 [1778.00–9601.20]	$5353.99 ± 6442.55 $3200.40 [1066.80–7 467.60]	2 vs. 1: < 0.001 3 vs. 1: < 0.001 3 vs. 2: < 0.001
Medical services	$3030.44 ± 2372.30 2515.45 [1483.66–3895.96]	$9148.58 ± 7 474.77 7022.74 [4948.60–10,655.50]	$8997.31 ± 6677.50 7191.72 [4325.52–11,438.92]	2 vs. 1: < 0.001 3 vs. 1: < 0.001 3 vs. 2: 0.814
Pharmaceutical services	$7108.08 ± 15,672.20 $1 603.78 $ [0–7 821.55]	$19,068.85 ± 28,697.93 $10,516.24 [693.10–26,259.20]	$18,275.41 ± 26,670.43 $8842.21 [350.17–27,464.87]	2 vs. 1: < 0.001 3 vs. 1: < 0.001 3 vs. 2: 0.745
Total costs	$94 433.68 ± 309 189.60 $10,601.97 [4384.47–36, 306.86]	$230 899.20 ± 514 675.30 $54,452.60 [20,972.53–22,6 549.70]	$191 248.40 ± 519 904.40 $37,681.20 [15,619.84–13,8 048.50]	2 vs. 1: < 0.001 3 vs. 1: < 0.001 3 vs. 2: 0.129

Inter‐group comparisons performed using the Kruskal–Wallis non‐parametric test.

## 4 Discussion

Using SA applied to long‐term administrative data, we identified three distinct care trajectories among adults in Quebec with MDs. The largest group (≈61%) showed minimal utilization of the healthcare system; the second group exhibited moderate utilization, mainly involving the GP and non‐psychiatrist specialists; while the smallest group was characterized by frequent psychiatric visits. These types differed in demographics and needs. Importantly, the mean 7‐year per‐patient costs ranged from about CAD 94,400 in Type 1 to CAD 230,900 in Type 2 (with Type 3 in between at CAD 191,200). In other words, although most patients have a low service utilization, a minority with higher‐intensity trajectories generate disproportionately high expenditures.

Since the study defined the index date as the date of the CCHS survey at which the respondent reported an MD, with a focus on five years before the index date and two years after it, this timeframe can have implications for the study's results. For a patient diagnosed long ago, the five preceding years may reflect chronic management or MD stability. This could partially explain the predominance of Type 1 (low users), who represent most of the sample. These individuals may be in remission or stable under medication (renewed by a GP), without requiring recent intensive care.

Our findings align in general with those of Kouakou et al. (2025), who applied latent class analysis (LCA) and latent profile analysis (LPA) methods to the same dataset. They identified four latent classes via LCA: (1) GP‐only users, (2) patients using a psychiatrist or with ≥1 ED visit/hospitalization, (3) those using other specialists, and (4) a “null utilization” class. They also performed LPA on counts of services, finding four medical‐service profiles (with mean annual counts of 41, 33, 7, and 12) and two hospitalization profiles (20% ever‐hospitalized). The broad story is similar: a large “null‐utilization” group coexists with smaller clusters distinguished by their pattern of use. For example, our Type  1 (61%) corresponds to their null‐utilization class, while our Types  2–3 roughly span their psychiatrist/ED versus specialist groups.

However, the classification schemes differ in detail due to methodological contrasts. Sequence analysis emphasizes the timing and order of events, whereas LCA and LPA group individuals based on overall counts or categories of use. In practice this meant our SA merged what LCA split: LCA's four classes collapsed into three trajectory types (LCA's classes 1 and 3 yield a group akin to our moderate‐use cluster). Moreover, LCA/LPA are parametric methods that estimate average patterns, which can be sensitive to the many zero‐use observations in the data. Sequence analysis, being nonparametric, required us to prioritize states (e.g., treating any hospitalization as higher‐priority than outpatient visits) but could capture complex sequences of care. These differences explain the slight divergence in results: our SA highlights the temporal structure (e.g., when care occurs in follow‐up), whereas latent models quantify overall service intensity. Neither approach is “right” or “wrong”—rather, they offer complementary views. In fact, all three methods (SA, LCA, and LPA) yielded broadly consistent insights, even though the exact number of groups and their composition varied.

The overall pattern we observe—a plurality of MD patients having very low service use, with a smaller number of higher‐intensity users—is consistent with previous findings in the literature. In diverse settings, trajectory studies in mental health typically identify multiple classes, with most patients belonging to a low‐use category. For instance, an Australian seven‐year study of women with mental health issues revealed six trajectories, among which a high‐user class represented only about 7% of the population (Dolja‐Gore et al., 2022). Studies of other psychiatric conditions in Quebec show comparable patterns. Vanasse et al. (2022) identified five healthcare utilization trajectories prior to schizophrenia diagnosis, with most participants in a minimal‐use class, and Behrendt‐Møller et al. (2019) found four trajectories among individuals with bipolar depression, again dominated by a low‐utilization group. Parikh et al. (1996) reported that approximately half of Ontarians with MD had no recorded health service use over a one‐year period. Altogether, our trajectory typology—predominantly minimal use coexisting with a few higher‐use patterns—mirrors the literature's depiction of heterogeneous and complex care pathways among people living with mood and mental health disorders.

Several strengths of our approach should be highlighted. The TorSaDE database offers a rich, long‐term view linking self‐reported diagnoses with 21 years of claims allowing it to capture a broad spectrum of care. Sequence analysis itself has the advantage of modeling the order of events, recognizing that, for example, a hospitalization followed by specialist visits is different from the reverse order. However, our study also has limitations that temper interpretation. First, we identified MD patients by self‐report in the survey. Self‐reported diagnosis has acceptable validity but differs from administrative diagnoses—in our cohort each source picked up different people. We chose self‐report to emphasize patient‐identified illness, but this could include some who never saw a doctor. Second, the administrative data are confined to publicly insured (RAMQ) services. That means any psychological counseling, private psychiatry, or community support outside RAMQ is unobserved. As many MD patients use such services, our trajectories represent only the covered portion of care. Third, SA has its own quirks: it is sensitive to long runs of “no contact” months and requires an a priori hierarchy of states (i.e., we gave priority to hospitalizations and emergency care). These analytic choices involve some researcher discretion. In short, data limitations (e.g., missing non‐RAMQ services) and methodological assumptions must be kept in mind when drawing conclusions.

Results indicate that the burden of the disease is unevenly distributed: a vulnerable minority (Type 3, ≈13%) accounts for a disproportionate share of specialized care resources, while the majority manages their condition with limited reliance on the public healthcare system. These findings suggest that patients with MDs should not be treated as a homogeneous group. Resource allocation should instead be tailored to the identified profiles. The Type 3 group (intensive psychiatric service users) appears socially vulnerable. They are more often single (42.2%), have low income (52.3% earning less than $20,000), and report poorer perceived mental health. For this subgroup, the study points to the need for intensive case management and enhanced support to prevent crises, rather than sporadic consultations. In contrast, for the majority (Type 1), heavy medical intervention is not necessarily warranted; integrated care models or community‐based support, which are less costly and better adapted, may be more appropriate. Moreover, low utilization of public services does not automatically indicate a failure of the healthcare system or the presence of access barriers. Appendix data (Table A1) show that individuals in Type 1 report better perceived mental health (43.7% rate it as “good” vs. 33.6% in Type 3) and lower levels of daily stress, suggesting a milder or more stable form of the disorder that may not require intensive care. Note that 27% of healthcare expenditures in Quebec are privately funded. It is therefore plausible that many Type 1 individuals rely on private psychologists or other non‐RAMQ services, which are not captured in administrative data, particularly given their relatively more favorable socioeconomic status compared with Type 3. Ultimately, the identified trajectory clusters and their cost profiles provide a more granular picture for healthcare planners: rather than treating all patients with MD as a single cohort, policymakers can allocate resources proportionally.

In summary, our SA adds temporal depth to the understanding of service pathways in MDs. It confirms that a diagnosis of MD does not automatically imply high service use, and it reveals the heterogeneous utilization within this population. The consistency of our findings with other trajectory studies suggests that mental health services must be flexible, recognizing diverse patient profiles. For future work, integrating data on non‐physician care (psychology, counseling, community programs) will be crucial to capture the full continuum of care. Nevertheless, the present results already inform healthcare system planners: identifying a large under‐served segment and quantifying the costs of intensive users should guide efforts to improve access and organize services effectively.

## 5 Conclusion

This study identified distinct care trajectories among adults with self‐reported mood disorders in Quebec using sequence analysis. Nearly 60% of individuals exhibited minimal or no use of publicly insured health services, while smaller subgroups showed moderate or intensive utilization through general practitioners, specialists, or hospital‐based care.

Overall, the results indicate that the burden of MDs is unevenly distributed across care trajectories. A relatively small and vulnerable subgroup concentrates a disproportionate share of specialized and costly services, whereas the majority manage their condition with limited reliance on the public healthcare system. These findings highlight the heterogeneity of health service utilization among individuals with MDs and show that a diagnosis does not systematically translate into high healthcare use. Importantly, the predominance of minimal users should be interpreted with caution, as it may reflect diverse needs, stable or milder forms of the disorder, and/or the use of privately funded or community‐based services not captured in administrative data, rather than unmet needs or access barriers alone.

The identification of distinct trajectory profiles and their associated cost gradients (ranging from CAD 94,400 to CAD 230,900 over 7 years) provides valuable insights for healthcare planning. Rather than treating patients with MDs as a homogeneous group, resource allocation and service organization should be tailored to the specific profiles identified. Finally, future research should integrate data on privately funded and community‐based care to better capture the full continuum of services used by people living with MDs.

## Author Contributions

ML and TGP were responsible for conceptualization, funding acquisition, project administration, resources, and supervision. MB and CRCK performed data curation and formal analysis. Investigation was conducted by MHN, MB, CRCK, ML, and TGP. Methodology was developed by MB, CRCK, ML, and TGP. All authors contributed to validation, visualization, writing – original draft, review and editing.

## Funding

This study was funded by the Unité Soutien SSA Québec.

## Conflicts of Interest

The authors declare no conflicts of interest.

## Data availability

Data are proprietary of the Institut de la statistique du Québec and cannot be shared.

## 1

**TABLE A1:** Comparison of descriptive characteristics between groups derived from SA.

Characteristics	Type 1 (*n* = 2714)	Type 2 (*n* = 1120)	Type 3 (*n* = 587)	*p*‐value
**Age, years**	48.6 ± 15.5	56.5 ± 15.3	49.3 ± 14.3	< 0.001
**Female, %**	56.6%	72.6%	60.4%	< 0.001
**Body mass index, kg/m^2^**	26.6 ± 5.71	28.2 ± 6.65	27.7 ± 6.03	< 0.001
**Education level, %**				< 0.001
Unknown	6.16%	3.21%	9.04%	
Incomplete secondary	20.6%	27.2%	15.7%	
High school diploma	14.8%	13.4%	11.1%	
Vocational/College diploma	25.2%	25.9%	26.8%	
Bachelor's degree	21.6%	19.4%	21.2%	
Graduate studies	11.7%	10.9%	16.3%	
**Marital status, %**				< 0.001
Married	27.1%	34.8%	20.8%	
Common‐law union	18.9%	17.2%	15.0%	
Widowed	3.83%	9.34%	2.97%	
Separated	4.42%	2.94%	4.80%	
Divorced	11.1%	15.8%	14.3%	
Single/Never married	33.6%	19.9%	42.2%	
**Annual household income, %**				< 0.001
Less than $20,000	20.4%	22.7%	27.5%	
$20,000 to < $40,000	23.8%	30.1%	23.0%	
$40,000 to < $60,000	18.7%	20.5%	19.2%	
$60,000 to < $80,000	13.5%	10.5%	13.6%	
$80,000 and more	23.7%	16.2%	16.7%	
**Annual respondent income, %**				< 0.001
Less than $20,000	42.7%	45.3%	52.3%	
$20,000 to < $40,000	27.5%	34.8%	24.2%	
$40,000 to <$60,000	15.2%	12.7%	16.0%	
$60,000 to <$80,000	8.94%	5.45%	5.00%	
$80,000 and more	5.65%	1.80%	2.60%	
**Country of birth, %**				0.383
Canada and North America	91.8%	93.2%	86.7%	
Central/South America	1.94%	1.46%	2.27%	
Europe	3.13%	3.58%	4.73%	
Asia and Oceania	1.87%	0.62%	2.85%	
Africa	1.28%	1.13%	3.55%	
**Perceived stress – Daily life, %**				0.530
Not at all stressed	6.40%	7.57%	5.38%	
Not very stressed	11.9%	12.6%	16.11%	
A little stressed	34.3%	32.7%	36.5%	
Quite stressed	35.6%	34.6%	29.0%	
Extremely stressed	11.9%	12.6%	13.1%	
**Perceived stress – Work, %**				0.316
Not at all stressed	5.02%	10.9%	4.82%	
Not very stressed	11.1%	9.37%	12.4%	
A little stressed	31.5%	28.3%	39.2%	
Quite stressed	38.0%	39.6%	34.6%	
Extremely stressed	14.3%	11.9%	8.95%	
**Perceived general health, %**				<0.001
Poor	6.06%	18.6%	11.20%	
Fair	21.9%	33.0%	30.3%	
Good	38.1%	29.1%	41.1%	
Very good	26.5%	16.9%	13.1%	
Excellent	7.43%	2.39%	4.30%	
**Perceived mental health, %**				<0.001
Poor	6.97%	7.16%	14.0%	
Fair	23.1%	23.5%	36.0%	
Good	43.7%	40.0%	33.6%	
Very good	19.5%	22.1%	12.0%	
Excellent	6.76%	7.22%	4.41%	
**Health‐related quality of life index (HUI‐3)** **^β^** **, score**	0.73 ± 0.27 (n = 1927)	0.59 ± 0.33 (n = 832)	0.67 ± 0.30 (n = 432)	<0.001

^β^ Score not available for individuals who completed the 2011–2012 cycle of the CCHS (*n* = 1111).
